# Book Reviews

**Published:** 1914-03

**Authors:** 


### BOOK REVIEWS.

*Books mentioned may be inspected at and ordered through this office. So far as possible, books received in any month will be reviewed in the issue of the second month following.*

Diagnostic Methods. Chemic, Bacteriologic and Microscopic. Ralph W. Webster, M. D., Ph. D., Chicago. 3d edition, 692 pages, 37 colored plates, 164 other illustrations; $4.50. P. Blakiston's Son & Co.

The speedy exhaustion of previous editions speaks well for the popularity of this book and the colored plates alone might well explain the popularity. While no one section, as the urine, gastric contents, fæces, parasitology, blood, etc., supplants special text books, the work in each section is thorough, up to the point of specialization, and, indeed, while the simpler tests are well treated, the average pracitioner will find in each section a larger program than he will probably carry out. This is wholesome, as it stimulates further study, and while it may not always be possible for the practitioner to follow the book to the limits thus set, there are frequently encountered cases in which it is worth while to make more elaborate investigations than the routine, and for these the book is an efficient guide. While aboundantly illustrated, the author does not depend upon illustrations to cover deficiencies of description, his style of writing being clear and interesting, inviting study in advance of the use of the book as a laboratory guide.

Clinical Pathology. P. N. Panton, M. A., M. B., B. C., London. P. Blakiston's Son & Co., Philadelphia. 446 pages, 11 colored plates, 2 uncolored, 45 other illustrations; $4.00.

It will be noted that this book covers somewhat the same subject as the preceding, but from a different viewpoint, as suggested by the titles. It is divided into the following sections: Blood, Bacteriology, Puncture Fluids, Urine, Alimentary System, Eye and Skin, Respiratory Tract, Histology. Considerable attention is given to bio-chemic blood reactions, diagnostic and therapeutic, to neoplasms generally, to preparation of tissues for microscopic examination, while the examination of excreta for purely diagnostic purposes, is covered more with reference to general pathologic principles than as bases for detailed diagnosis. At the same time the designation of the field of pathology as "clinical" is amply justified by the practical way in which the author treats each section.

---

The Practical Medicine Series. 10 annual volumes, under the general editorial charge of Charles L. Mix, A. M., M. D., Chicago; series of 1913. The Year Book Publishers, Chicago; price for series, $10.00.

---

Vol. 9, Skin and Venereal Diseases: Miscellaneous Topics. Edited by W. L. Baum and Harold N. Moyer, Chicago: 228 pages, illustrated; $1.35.

Vol. 10, Nervous and Mental Diseases. Edited by Hugh T. Patrick and Peter Bassoe, Chicago; 244 pages, illustrated; $1.35.

These two volumes complete the series for 1913, the first volume of the 1914 series to appear shortly. Each volume is complete in itself, but we strongly advise subscription to the entire series. As a review of current literature, brought up to within a few weeks of date of publication of each volume, this series is unrivaled, and, for economy and completeness, maintains the highest standards established for such reviews.

---

Pathognomy of Pain. Pamphlet of 26 pages, copiously illustrated, published by the Arlington Chemical Co. of Yonkers, N. Y., distributed to physicians free.

This gives plates in colors showing distribution of cranial, cervical, dorsal, lumbar and sacral sensory roots, a list of segments supplying the various viscera, diagnostic tables as to distribution and other characteristics of pain. It is a very valuable summary of sensory anatomic distribution and of the diagnostics of painful sensations.

---

The Champlain Tercentenary. Final Report, 1913. Prepared by Henry Wayland Hill, LL. D., Secretary of the Commission; Albany, J. B. Lyon Co., State Printers.

This is an imposing volume of 325 pages, copiously illustrated. The first part deals with the construction of memorials to Champlain and the visit of the French delegation to America; the second with the dedicatory ceremonies at various places of historic interest about Lake Champlain in 1912; the third with the part played by the U. S. Government, the history of the work of the commission, and general historic and biographic data; while the appendix contains the report of the House Committee on Foreign Affairs, a description of historic English forts, the original report of Capt. Mott regarding the appointment of Benedict Arnold to command the Ticonderoga expedition, notes on the archæology of the Champlain valley by Prof. Geo. H. Perkins, State Geologist of Vermont, and financial statements, the whole book being indexed. The extreme importance of the commemoration of the history of this region cannot be too strongly emphasized, and it is well that the State has empowered a Commission not only to represent it in the arrangement of details and in showing hospitality to distinguished foreign guests, but capable of recording history. This report, as well as the former volume, reflects credit especially upon the Secretary of the Commission.

---

THE TREATMENT OF RHEUMATIC INFECTIONS. Press of Parke, Davis & Co., Detroit. 134 pages, frontispiece, plate of pure culture of streptococcus rheumaticus sent free to physicians on request.

This book contains a general description of the use of Phylacogens, methods of preparation, testing, technique, etc., as well as bibliography, extracts from clinical reports, index, etc. Contraindications are frankly stated. In a total of 6324 cases, 83 per cent. recovered, 17 are recorded as failures, but include cases in which rheumatism in the specific case did not exist, moribund cases and the like. In no case could death be attributed to the use of phylacogen, but a considerable list of fatal cases is given, including syphilis, cancer, advanced tuberculosis, sarcoma, typhoid fever, puerperal and other forms of sepsis, etc. All things considered, few, if any, remedies have given such brilliant results and have been so free from danger if used with any sort of discrimination.

---

A TEXT-BOOK OF THE PRACTICE OF MEDICINE. Eleventh edition, thoroughly revised, by James M. Anders, M. D., Ph. D., LL. D., Professor of Medicine and Clinical Medicine, Medico-Chirurgical College, Philadelphia. Octavo of 1335 pages, fully illustrated. Philadelphia and London: W. B. Saunders Company, 1913. Cloth, $5.50 net; half Morocco, $7.00.

The value of this book is impressed by the number of the edition. No attempt has been made to depart from conventional arrangements or, in other ways, to gain a show of novelty. On the other hand, the most recent advances have been included and the work has been rewritten as necessary. It is a substantial, thorough treatise deserving a continuance of the favorable regard of the profession.

---

Materia Medica, Pharmacology, Therapeutics and Prescription Writing, for Students and Practitioners. By Walter A. Bastedo, Ph. G., M. D., Associate in Pharmacology and Therapeutics at Columbia University. Octavo of 602 pages, illustrated. Philadelphia and London: W. B. Saunders Company, 1913. Cloth, $3.50 net.

The author has taken as a model the old fashioned systematic treatise, classifying therapeutics according to general methods of action, instead of cutting the Gordion knot by recourse to an alphabetic arrangement. It is obvious that the more discriminating our knowledge of pharmacology becomes, the more difficult is it to follow the older system, yet it has advantages that are equally plain. The author has most skilfully avoided being a slave to a system of classification, while preserving the merits of this system. It would be a gross injustice to stigmatize the book itself as old fashioned. Rearrangements of old tables of classification, addition of drug groups, definite scientific statements where we formerly had only general impressions, sometimes very crude and even incorrect, the addition of many valuable remedies and even of whole groups of therapeutic measures, the solution of therapeutic problems formerly insoluble, and, more valuable yet, wholesome skepticism and even denial of therapeutic powers formerly believed in, mark a tremendous advance over earlier text books.

---

Mortality Statistics, Bureau of the Census, 1911.

Great improvement is shown both in the original detailed reports and in the scientific assortment and compilation. The relative fewness of unspecified ages, dubious diagnoses, etc., show that we have, for the registration area, a mass of carefully compiled, statistics dependable for scientific conclusions, barring, always, the personal error of individual reporters.

---

Anatomy and Physiology, a Text Book for Nurses. John Forsyth Little, M. D., Philadelphia, published by Lea & Febiger; 483 pages, 149 engravings and 4 plates; $1.75.

This is one of the Nurses' Text Book Series. The customary arrangement of anatomies is followed, including a brief preliminary consideration of histology and embryology, but physiology, including certain parts of chemistry, is interpolated at convenient points in the descriptions so that the student is impressed with the use of the organs and, one might also say, the excuse for learning dry details of structural arrangement, as she progresses. It has seemed to us that this might be a good way to teach medical students also, and, indeed, our own study of these branches in the medical school was facilitated by having had what was, at the time, an exceptionally thorough course in physiology at a high school. The book concludes with an exceptionally good glossary and an index.

---

PRINCIPLES OF SURGERY. By W. A. Bryan, A. M., M. D., Professor of Surgery and Clinical Surgery at Vanderbilt University, Nashville, Tennessee. Octavo of 677 pages with 224 original illustrations. Philadelphia and London: W. B. Saunders Company, 1913. Cloth, $4.00 net.

The writer well states that the title of his book might be limited to the word "Principles," as it enters broadly into pathology and other branches of medicine, though always, apparently, with a view to their bearing on surgery. About half the book deals with what might be termed surgical pathology, including sepsis, asepsis and certain infections, as tuberculosis, syphilis, actinomycosis, etc., and about a third with tumors. While treatment is considered under each appropriate division and a chapter is devoted to anæsthesia, the author has avoided competition with works on operation and surgical technic. On the other hand, he has given a practical turn to the discussion of principles, so as to avoid duplicating text books on pathology. In short, he has struck a happy mean which has, for some time, not been represented in text books.

---

HISTORY OF MEDICINE, WITH MEDICAL CHRONOLOGY, BIBLIOGRAPHIC DATA AND TEST QUESTIONS, by Fielding H. Garrison, A. B., M. D., Principal Assistant Librarian, Surgeon General's Office, Washington, D. C., Editor of the "Index Medicus," Octavo of 763 pages, many portraits. W. B. Saunders Company, Philadelphia and London, 1913. Cloth, $6.00 net; half Morocco, $7.50 net.

The ancient Egytian, Oriental and Greek (including Roman) and other early systems are first considered, and their essential identity is pointed out. More than half of the book is devoted to the nineteenth century, biography and medical progress being jointly considered. The appendix on medical chronology, with general historic events as landmarks, is especially valuable. Except brief references, the author wisely omits biographies of living men. 150 test questions, in an appendix, serve to call the attention of the reader to salient points and to render his study more thorough.

---

Proceedings of the Royal Society of Medicine, Vol. 7, No. 2, December, 1913. Published by Longmans, Green & Co., London, New York, etc. 7/6d.

Contrary to our precedent in regard to works of this nature, covering a wide variety of monographs, representing the work of different sections, in the present instance, we glean the following points of special interest:

Chaldecott and Bryan speak highly of Crile's technic to insure innocuous association in operations. Albert Wilson, from an experience of about forty cases since 1880, recommends the excision of the primary lesion of syphilis, as diminishing the degree of infection and even eradicating the disease. He also praises arsacetin, especially in tertiary cases and is conservative in regard to the diagnostic reliance to be placed on the Wassermann reaction.

Gossage and Hicks report two cases of non-cancerous gastric tumor, aged 50 and 37, both fatal, apparently on account of anæmia, 20 per cent. Hb, 1,560,000 reds, 24,200 whites and 25 per cent. Hb., 1,240,000 reds and 39,000 whites respectively. The first tumor was of the size of a golf ball, 2 inches from the pylorus, sessile, fibro-leio-myoma; the second, same size, attached by pedicle to middle of posterior wall, fibro-sarcoma of slight malignancy.

Pitt reports obstruction of Steno's duct by calcified fish bone, and alludes to other cases in the literature, 27 in Wharton's, 19 in Steno's and 2 in sublingual ducts.

Crowe details cultural experiments and concludes that the essential cause of rheumatoid arthritis is the common inhabitant of the skin, the micrococcus epidermidis, variety deformans, producing the disease mainly by action on some portion of the nervous system and often associated with other germs.

Minett, from a careful study of the micrococcus neoformans, isolated from non-ulcerating cancers, concludes that this is merely a variety of staphylococcus albus due to deep growth, such as can be isolated from deep tissues in both human beings and animals, the modification being due to anærobic conditions, and that there are no biologic reactions indicating any specific relation to cancer.

Coxa Vara, Its Pathology and Treatment. R. C. Elmslie, M. S., F. R. C. S., London, Oxford University Press, American Branch, 35 W. 32, New York; 35 pages, 34 radiograms, photographs and cuts; paper cover, 60 cents.

This is a valuable monograph, both didactic and clinical.

---

Report of the Committee on Inquiry Into the Departments of Health, Charities and Bellevue and Allied Hospitals in the City of New York.

Section 7—Care of Out-Patients. This report will interest all engaged in analogous lines of medical service and sociology. It is frankly critical at times, as it should be, since the ultimate responsibility for shortcomings rests with the city. The third section on sickness in the home and proposed health center is especially interesting. Naturally, the report, so far as details go, applies more particularly to problems of the Metropolis and only in a general way to those of smaller cities and towns like those of our special territory.

---

Transactions of the American Laryngologic, Rhinologic and Otologic Society of 19th Annual Meeting, held in Washington, May 8-10, 1913.

Transactions of the American Laryngologic Association of 35th Annual Meeting, held in Washington, May 5-7, 1913.

Both of these volumes are published by the organizations and contain illustrated monographs of great value. In accordance with our custom, we do not select any special papers for comment, as it is obviously unfair to discriminate and impossible to do justice to all. As many individuals are members of both organizations, and as the dates of meetings have obviously been selected to allow attendance at both, we venture to suggest that the maintenance of essentially duplicate organizations in this and other specialties is contrary to general principles of economics.

---

The Pathology of Growth: Tumors. By Charles Powell White, M. D., F. R. C. S., Manchester, Eng., published by Paul B. Hoeber, 69 E. 59, New York; 235 pages, 86 illustrations; $3.50.

This is the first volume of a series under the general editorship of A. E. Boycott, B. S., M. A., M. D., a name more familiar to us as a common noun or verb than in its original use as a proper noun. The present volume discusses the relation of tumors to growth in the general sense, including hypertrophy and the characteristics of each particular neoplasm. While the morbid anatomy and physiology are well treated, the chief value of the work lies in its scholarly discussion of general principles of tumor development, ætiology, etc. We commend it highly, not only to the pathologist, but to the general practitioner as containing interesting information not usually found in text books nor readily attained elsewhere, and as suggestive of lines of both laboratory and clinical research, some of which may throw important light on a vaguely understood subject. It is a work that makes the reader think, without being a purely speculative presentation of the subject.

---

Diseases of the Kidneys and Nervous System. By A. L. Blackwood, B. S., M. D., Professor of Clinical Medicine in the Hahnemann Medical College, Chicago. Author of "A Manual of Materia Medica, Therapeutics and Pharmacology," "Diseases of the Heart," "Diseases of the Lungs," "Diseases of the Liver, Pancreas and Ductless Glands," "The Food Tract, its Ailments and Diseases of the Peritoneum" and "Contagious and Constitutional Diseases." 346 pages; cloth, $1.50; postage, 9 cents. Philadelphia. Boericke & Tafel, 1913.

This is the sixth and final volume in a series covering internal medicine, by the same author, but is independent of the other volumes. As stated by the author, the work is intended for the student and general practitioner, not for the specialist. The common diseases are briefly discussed, without entering into minutiæ or problems sub judice. Reference is facilitated by such headings, in black type, as Definition, Etiology, Pathology, etc. Drug treatment is mainly limited to mention of appropriate homeopathic remedies.

